# Metabolic-driven analytics of traumatic brain injury and neuroprotection by ethyl pyruvate

**DOI:** 10.1186/s12974-024-03280-8

**Published:** 2024-11-14

**Authors:** Nikita Golovachev, Lorraine Siebold, Richard L. Sutton, Sima Ghavim, Neil G. Harris, Brenda Bartnik-Olson

**Affiliations:** 1 School of Medicine, Loma Linda University, 11175 Campus St, Loma Linda, CA 92350 USA; 2 David Geffen School of Medicine, Neurotrauma Laboratory, University of California Los Angeles, 58-125 CHS, 650 Charles E. Young Dr. S., Los Angeles, CA 90095 USA; 3 Department of Radiology, Loma Linda University, 11234 Anderson St, Room B623 MRI, Loma Linda, CA 92354 USA

**Keywords:** Traumatic brain injury, Controlled cortical impact, Oxidative stress, Inflammation, Metabolomics, Liquid chromatography–mass spectrometry, Gas chromatography–mass spectrometry

## Abstract

**Background:**

Research on traumatic brain injury (TBI) highlights the significance of counteracting its metabolic impact via exogenous fuels to support metabolism and diminish cellular damage. While ethyl pyruvate (EP) treatment shows promise in normalizing cellular metabolism and providing neuroprotection, there is a gap in understanding the precise metabolic pathways involved. Metabolomic analysis of the acute post-injury metabolic effects, with and without EP treatment, aims to deepen our knowledge by identifying and comparing the metabolite profiles, thereby illuminating the injury's effects and EP's therapeutic potential.

**Methods:**

In the current study, an untargeted metabolomics approach was used to reveal brain metabolism changes in rats 24 h after a controlled cortical impact (CCI) injury, with or without EP treatment. Using principal component analysis (PCA), volcano plots, Random Forest and pathway analysis we differentiated the brain metabolomes of CCI and sham injured animals treated with saline (Veh) or EP, identifying key metabolites and pathways affected by injury. Additionally, the effect of EP on the non-injured brain was also explored.

**Results:**

PCA showed a clear separation of the four study groups (sham-Veh, CCI-Veh, sham-EP, CCI-EP) based on injury. Following CCI injury (CCI-Veh), 109 metabolites belonging to the amino acid, carbohydrate, lipid, nucleotide, and xenobiotic families exhibited a twofold change at 24 h compared to the sham-Veh group, with 93 of these significantly increasing and 16 significantly decreasing (*p* < 0.05). CCI animals were treated with EP (CCI-EP) showed only 5 metabolites in the carbohydrate, amino acids, peptides, nucleotides, lipids, and xenobiotics super families that exhibited a twofold change, compared to the CCI-Veh group (*p* < 0.05). In the non-injured brain, EP treatment (sham-EP) resulted in a twofold change in 6 metabolites within the amino acid, peptide, nucleotide, and lipid super families compared to saline treated sham animals (sham-Veh, *p* < 0.05).

**Conclusions:**

This study delineates the unique metabolic signatures resulting from a CCI injury and those related to EP treatment in both the injured and non-injured brain, underscoring the metabolic adaptations to brain injury and the effects of EP. Our analysis uncovers significant shifts in metabolites associated with inflammation, energy metabolism, and neuroprotection after injury, and demonstrates how EP intervention after injury alters metabolites associated with mitigating inflammation and oxidative damage.

**Supplementary Information:**

The online version contains supplementary material available at 10.1186/s12974-024-03280-8.

## Background

Traumatic brain injury (TBI) is one of the leading causes of death and disability in the US and worldwide [1]. The consequences of injury are separated into two distinct categories with each having its own pathophysiology. Primary injury refers to the immediate damage inflicted on the brain due to an external force, whereas secondary injury involves a series of pathological processes triggered post-injury, encompassing oxidative stress and activation of inflammatory pathways [2]. There is an increase in energy demand and hyperglycolysis in the early stage (minutes to hours) followed by a more prolonged period of hypometabolism consisting of a reduced metabolic rate of glucose metabolism [3, 4]. In previous studies assessing the metabolic fate of glucose following a controlled cortical impact (CCI), increased levels of glucose present in the brain at 24 h suggested that the hypometabolic period is associated with impaired glucose uptake, metabolism, and low neural activity [5, 6]. Furthermore, this hyperglycemia can reduce cerebral blood flow and also promote the production of reactive oxygen species mediated by NADPH oxidase exacerbating the ongoing oxidative stress [7, 8].

TBI induces a robust inflammatory response that involves not only resident brain cells but also the recruitment of peripheral immune cells, and the infiltration of these cells into the brain exacerbates the inflammatory response [9]. Following brain injury, reduced oxygen consumption due to mitochondrial dysfunction and decreased activities of complex I and cytochrome oxidase (complex IV) result in an electron buildup, significantly contributing to the production of reactive oxygen species [10–12]. Microglia, upon activation post-TBI, release proinflammatory cytokines such as TNF-α, which can have both damaging and protective effects on the brain tissue [13]. Additionally, astrocytes are activated in response to TBI and contribute to the neuroinflammatory milieu by producing cytokines and chemokines [14].

One treatment strategy focuses on attenuating the energy deficit after injury. Studies have examined the benefit of exogenous fuel administration after TBI, particularly focusing on glucose metabolic pathways and the downstream products of its metabolism [3, 5]. This treatment is an attempt to ameliorate ATP depletion and reduce deficits in metabolic pathways, thereby preventing cell death.

Recent attention has been paid to pyruvate and its neuroprotective effects after injury. Pharmacologic administration of sodium pyruvate has the ability to cross the BBB and increase the activity of the TCA cycle [15, 16]. Studies have demonstrated that it is effective in scavenging reactive oxygen species (ROS) and hydrogen peroxide, showcasing advantageous antioxidant properties and improved mitochondrial stability after TBI [17, 18]. However, the pyruvate anion has poor stability in aqueous solution and can undergo a condensation reaction that reduces metabolic activity. Ethyl pyruvate (EP) is an ester formed from pyruvate and ethanol that can function as an ROS scavenger and has also been shown to have anti-inflammatory effects. In contrast to the anion form of pyruvate, the lipophilic nature of EP allows it to penetrate biological membranes more readily, improving passage through the BBB without the use of monocarboxylate transporters [19, 20]. The electrically neutral properties of EP also allow it to have greater potency than pyruvate on a molar basis.

Research using animal models is beginning to reveal the diverse positive impacts of EP following TBI. Notably, EP administration has been shown to decrease the density of microglia in the ipsilateral cortex after TBI, indicating a reduction in inflammation [21]. EP treatment also attenuated the TBI-induced deficit in mitochondrial cytochrome oxidase, reducing the oxidative stress that follows mitochondrial dysfunction [21]. Finally, EP was shown to enhance cerebral glucose utilization and exert a neuroprotective effect after injury [21]. Despite data showing a reduction in the inflammatory response and a significant normalization of cellular metabolism and associated neuroprotection, there have been no studies that have identified the metabolic pathways that may contribute to the beneficial effects on cell survival after EP treatment.

The goal of this work is to expand the understanding of the metabolic pathways impacted by TBI by identifying changes in the brain metabolome induced by injury and comparing these to the metabolic profiles observed in the non-injured brain. We reasoned that a brain metabolomics approach acutely after TBI would provide more comprehensive information about injury status and inflammation based on a signature of markers, rather than a single biomarker. This knowledge could then be used to inform future treatments more precisely to the unique metabolic pathways that are impacted by injury. Moreover, we believe that this approach would allow for a broader understanding of the metabolic impact of injury beyond just the commonly studied glucose pathways at this early-stage post-injury. Based on existing evidence on the metabolic effects of EP, we hypothesize that its administration will not only boost free radical detoxification via increased glutathione and other reducing agents but also enhance oxidative metabolism by elevating acetyl CoA levels, while concurrently mitigating inflammation through downregulation of pro-inflammatory cytokines and modulation of key inflammatory pathways.

## Methods

### Injury and tissue collection

All procedures were conducted with approval by the UCLA Chancellor’s Committee for Animal Research. Thirty adult Sprague–Dawley male rats (305 ± 2.3 g) from Charles River Breeding Labs (Hollister, CA, USA) were randomized into four groups via a random numbers table: sham vehicle (saline)-treated (sham-Veh, *n* = 6), moderate CCI vehicle-treated (CCI-Veh, *n* = 9), sham EP-treated (sham-EP, *n* = 6), and CCI EP-treated (CCI-EP, *n* = 9). Sample sizes were based on our previous studies showing significant neuroprotection, improved cerebral glucose utilization, and improved neurobehavioral outcome following EP treatment [6, 21].

Rats were pair-housed and allowed to acclimate for 1–2 weeks prior to injury, with food and water available ad libitum and room-controlled temperature and lighting (70–76 °F, 30–70% humidity, 12 h light on/off schedule). Rats were anesthetized with isoflurane (2.0–2.5% in 100% O_2_, flow rate = 2.0 ml/min) followed by placement in a stereotaxic frame (Kopf instruments, Tujunga, CA, USA). Aseptic conditions and stable body temperature were maintained throughout the surgical procedures. Body temperature of 37 ± 1 °C in the rats was achieved through a thermostatically controlled heating pad (Harvard Apparatus, Holliston, MA, USA). Following anesthesia, a midline incision was made to allow for reflection of the skin, fascia, and temporal muscles to expose the underlying skull. Rats in the CCI group received a 6-mm craniectomy over the left parietal cortex, centered 3-mm posterior and 3.5-mm lateral to bregma. For the cortical injury, an electronically controlled, small bore, dual-stroke pneumatic piston cylinder (Hydraulics Control, Inc., Emeryville, CA, USA) containing a circular, flat-tipped impactor was mounted onto a stereotaxic micro-manipulator, angled at 20° from vertical and centered within the craniectomy. The CCI rats received a cortical impact at a depth of 2.0 mm (~ 2.2 m/s velocity, 20 psi, duration = 250 ms) resulting in a moderate severity injury. Sham rats underwent similar procedures without the impact. Following injury, the scalp was sutured, and bupivacaine (0.1–0.14 mg/kg, subcutaneous) was injected around the incision site. Rats recovered in a heated cage (36–38 °C) until ambulatory and then were returned to their home cage. Vehicle alone (0.1 M phosphate buffered saline) or EP (40 mg/kg, i.p.) was administered at 0, 1, 3 and 6 h post-injury [3, 6, 21]. The treatment approach utilized was informed by research indicating that energy requirements following CCI injury remain elevated for at least 2 h after injury [22], and that the occurrence of post-TBI depolarization and neuronal hyperexcitability could extend the period of imbalance between fuel supply and energy demands [23–27]. At 24 h post-injury animals were euthanized by decapitation under isoflurane anesthesia (3–3.5% in 100% O_2_, flow rate = 3.0 ml/min) and tissue samples were collected from the left cortex (~ 4mm^2^) and frozen/powdered in liquid nitrogen.

### Metabolomics analysis

Samples were shipped to Metabolon, Inc. (Durham, NC, USA), where tissue samples were extracted and prepared for analyses of global metabolic profiles using Ultra Performance Liquid Chromatography–Tandem Mass Spectrometry (UPLC–MS/MS) or Gas Chromatography–Mass Spectrometry (GC–MS) following Metabolon's standard solvent extraction method (https://www.metabolon.com/support/portal/experimental-procedures/). The extracted samples were split into equal parts for GC–MS and LC–MS/MS analysis. For GC–MS, samples were derivatized using bistrimethyl-silyl-triflouroacetamide and analyzed on a Thermo-Finnigan Trace DSQ fast-scanning single-quadrupole mass spectrometer using electron impact ionization and operated at unit mass resolving power. UPLC-MS/MS analysis was performed on a Thermo Scientific Q-exactive high resolution/accurate mass spectrometer interfaced with a heated electrospray ionization source and Orbitrap mass analyzer operating at 35,000 mass resolution [28].

### Data filtering and analysis

The UPLC-MS/MS and GC–MS analysis yielded 503 unique metabolites. Data filtering included missing value imputation of any metabolite that was not detected in individual samples but detected in other samples using small value replacement, where all missing values were replaced with 1/5th the minimum value (n = 124 or 1.2%). Additionally, metabolites with more than 50% missing values in any one group were excluded from further analysis (n = 49) as they were considered to have a concentration below the UPLC-MS/MS or GC–MS limit of detection. Of the 454 remaining metabolites, only those with metabolites with a corresponding Human Metabolome Database (HMDB) identification (n = 355 metabolites) underwent subsequent log transformation and multivariate analysis. Principal component analysis (PCA) was used to evaluate unsupervised separation of the metabolic profile between all four groups using MetaboAnalyst 6.0 [29]. Significantly changing metabolites resulting from CCI injury (sham-Veh and CCI-Veh), the effect of EP on the injured brain (sham-EP and CCI-EP), and the effect of EP on the uninjured brain (sham-Veh and EP-Veh) were determined using a threshold fold change of 2.0 with a two-sample Student’s t-test (*p* < 0.05) and visualized using Volcano plots. A twofold change was selected based on a prior study employing a similar untargeted metabolomics approach in a rodent model of TBI [30]. The Random Forest analysis (RFA) module in MetaboAnalysis was used as an additional feature selection tool to help identify which metabolites were most significant in distinguishing between the different groups. The model was constructed using 5,000 decision trees to ensure model stability. At each node, the number of predictors tested was optimized to 7 to ensure overfitting. To ensure reproducibility, a constant seed (123,456) was set prior to model construction. The model’s performance was assessed and optimized using out-of-bag error estimates. Pathway analysis for each comparison was conducted on the combined metabolites identified in the RFA variable importance plot (VIP) and the significant metabolites identified in the volcano plots. To conduct pathway analysis, selected metabolites with HMDB identifiers found within the Rattus norvegicus pathway library were analyzed using the MetaboAnalyst Globaltest pathway enrichment analysis method and relative betweenness centrality node [29, 31]. MetaboAnalyst and Prism Graph Pad (Version 9.2) were used for statistical analysis and graphing.

## Results

**Fig. 1:**
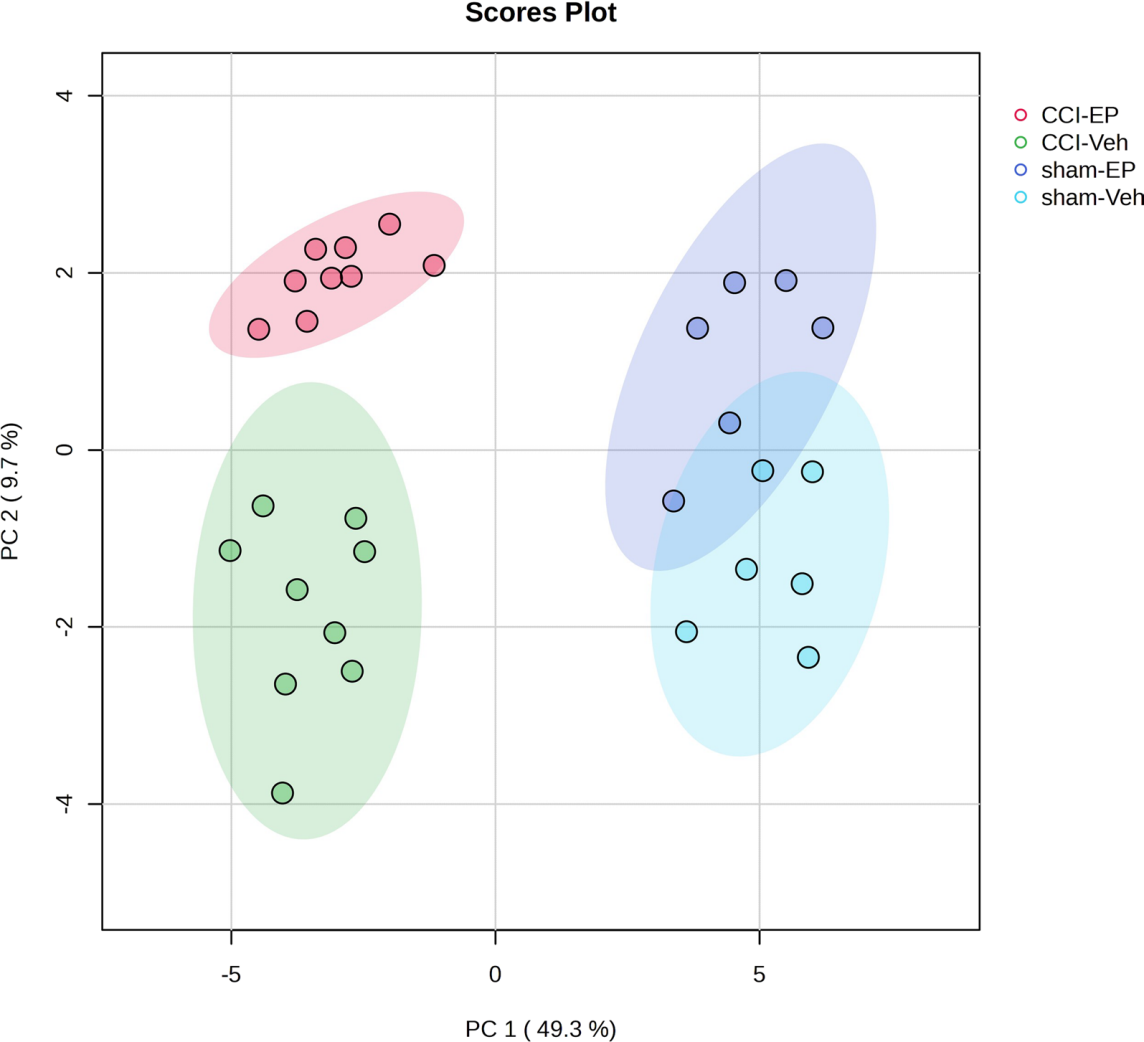
2D PCA scores plot with 95% confidence ellipses showing the effect of injury and EP treatment on the brain metabolome. A clear injury effect is noted by the separation of groups in PC1. A less robust treatment effect is seen in the injured brain as a separation of groups in PC2. There is no effect of EP in the non-injured brain as the sham-EP and sham-Veh groups overlap in PC2

Figure 1 shows the 2D PCA scores plot with 95% confidence ellipses of the four groups included in our study. PC1 and PC2 account for 49.3% and 9.7% of the total variance, respectively. Sham-Veh samples and sham-EP cluster on the positive side of PC 1 whereas CCI-Veh and CCI-EP samples cluster on the negative side of PC1, demonstrating the primary effect of injury. The effect of EP on the CCI injured brain is defined by the separation of the CCI-EP and CCI-Veh groups in PC 2. The metabolic effect of EP on the non-injured (sham) brain is not significant as sham-EP and sham-Veh groups overlap in PC2.

### CCI-induced changes in the brain metabolome

**Fig. 2:**
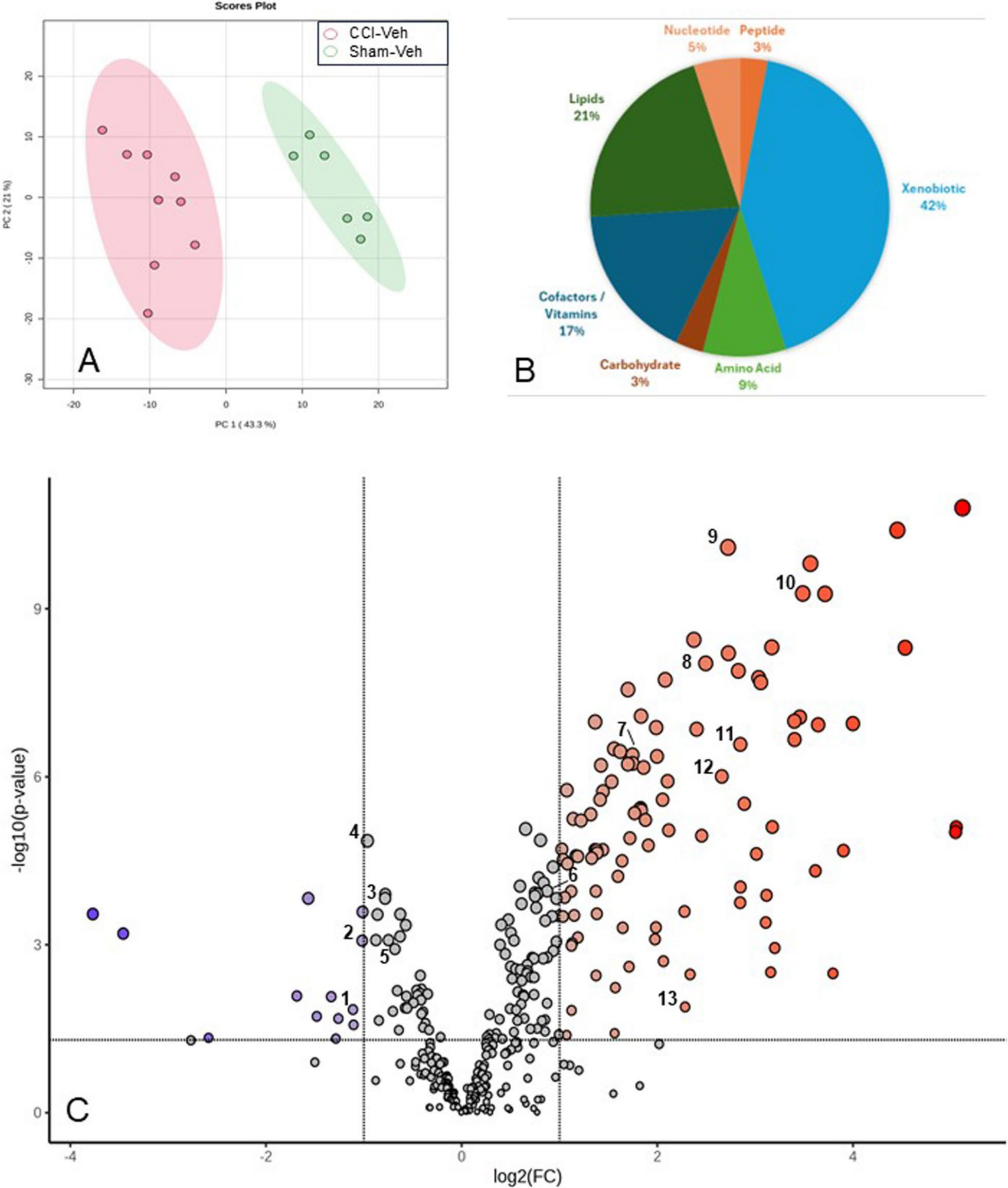
Profound changes in metabolism following TBI. **A** PCA with 95% confidence ellipses demonstrating a clear separation between the sham-Veh and CCI-Veh groups. **B** Pie chart showing the percentage of altered metabolites in each metabolic super family. Amino Acids composed the largest portion of the significantly altered metabolites with 43% of the amino acid metabolites significantly changing. **C** Volcano plot of the metabolites showing a twofold change in concentration following injury (*p* < 0.05). Numbered points indicate key metabolites identified in the Random Forest and pathway analysis associated with injury induced changes in metabolism (see Fig. 8). 1. cysteine; 2. glutathione disulfide; 3. GABA; 4. acetyl CoA; 5. homocarnosine; 6. alpha-ketoglutarate; 7. 4-acetoamidobutanoate; 8. glucose; 9. hypotaurine; 10. putrescine; 11. cystathionine; 12. opthalmate; 13. agmantine

PCA identified a clear metabolic distinction between the CCI-Veh and sham-Veh groups, with the first two principal components accounting for 57.5% of the variability (Fig. 2A). This metabolic profile of the injured brain featured significantly altered metabolites in the amino acid, nucleotide, lipid, carbohydrate, peptide, cofactors/vitamins, and xenobiotic metabolite families, with most metabolites falling into the amino acid and nucleotide super pathways (Fig. 2B). The volcano plot identified 102 metabolites showing a twofold change, with 93 metabolites significantly increased and 14 metabolites significantly decreased in the CCI-Veh group compared to sham-Veh (*p* < 0.05, Fig. 2C).

### Effect of EP treatment on the CCI injured brain

**Fig. 3:**
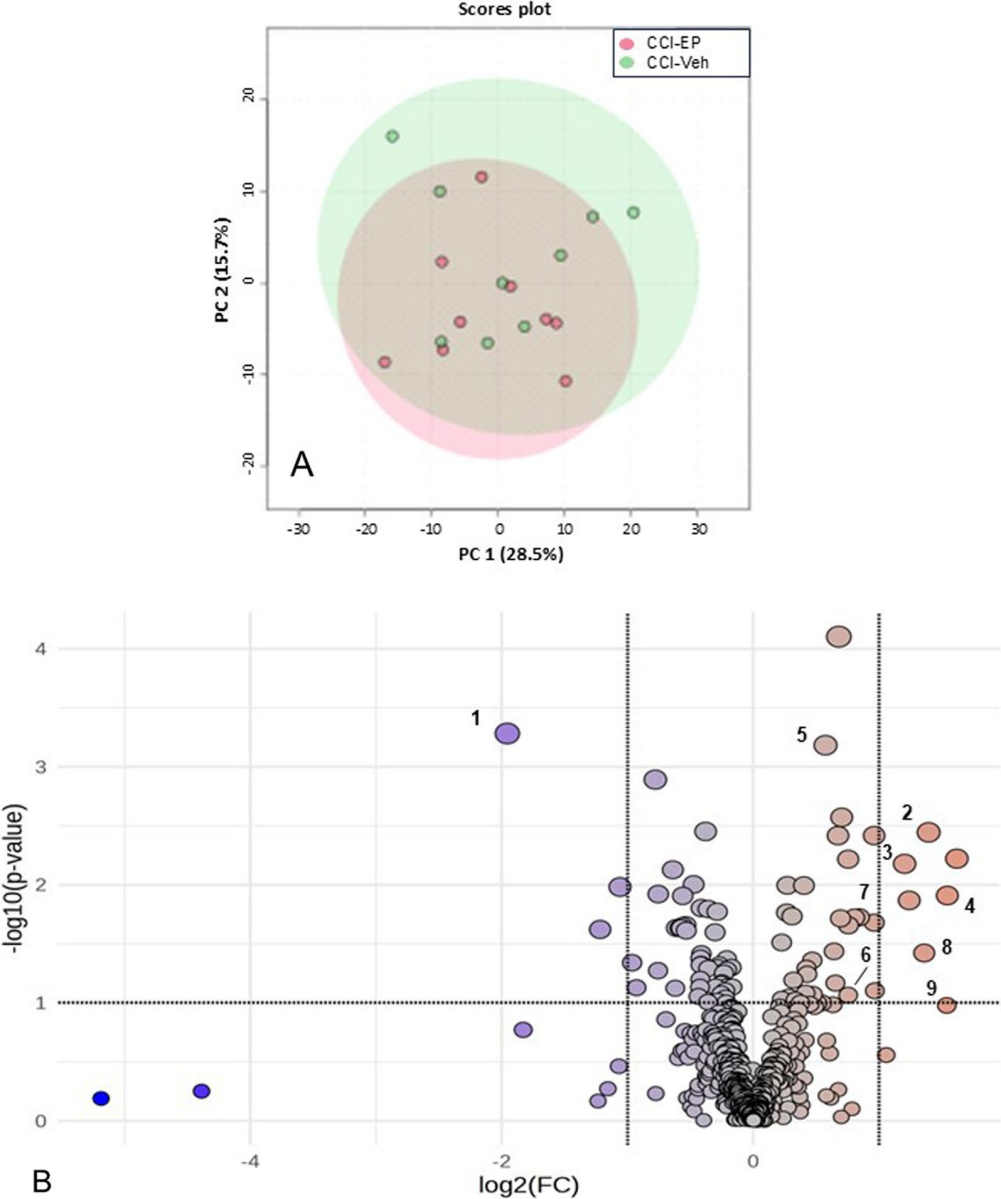
Effects of ethyl pyruvate treatment in the injured brain. **A** PCA with 95% confidence ellipses showing an overlapping metabolic profile between CCI-EP and CCI-Veh groups. **B** Volcano plot of the 5 metabolites showing a twofold change in concentration after treatment (*p* < 0.05). Numbered points indicate key metabolites identified in the Random Forest and pathway analysis following EP treatment in the injured brain (see Fig. 12). 1. cystine; 2. adenosine; 3. adenosine 5-monophosphate; 4. ascorbate; 5. acetyl CoA; 6. cysteine; 7. glutathione disulfide; 8. phosphoenolpyruvate; 9. 2-phosphoglycerate

PCA comparing the metabolic effect of EP treatment following CCI injury (CCI-EP) to the CCI-Veh group showed overlapping metabolite profiles, with the first two principal components accounting for 44.2% of the variability (Fig. 3A). The volcano plot identified a twofold increase in adenosine, adenosine 5ʹ-monophosphate (AMP), ascorbate (Vitamin C), and nicotinamide ribonucleotide (NMN) concentration and a twofold decrease in cystine metabolite concentration in the CCI-EP group compared to the CCI-Veh group (Fig. 3B). The affected metabolites belonged to the amino acid, cofactor and vitamin, and nucleotide super families.

### Effect of EP treatment on the non-injured brain

**Fig. 4:**
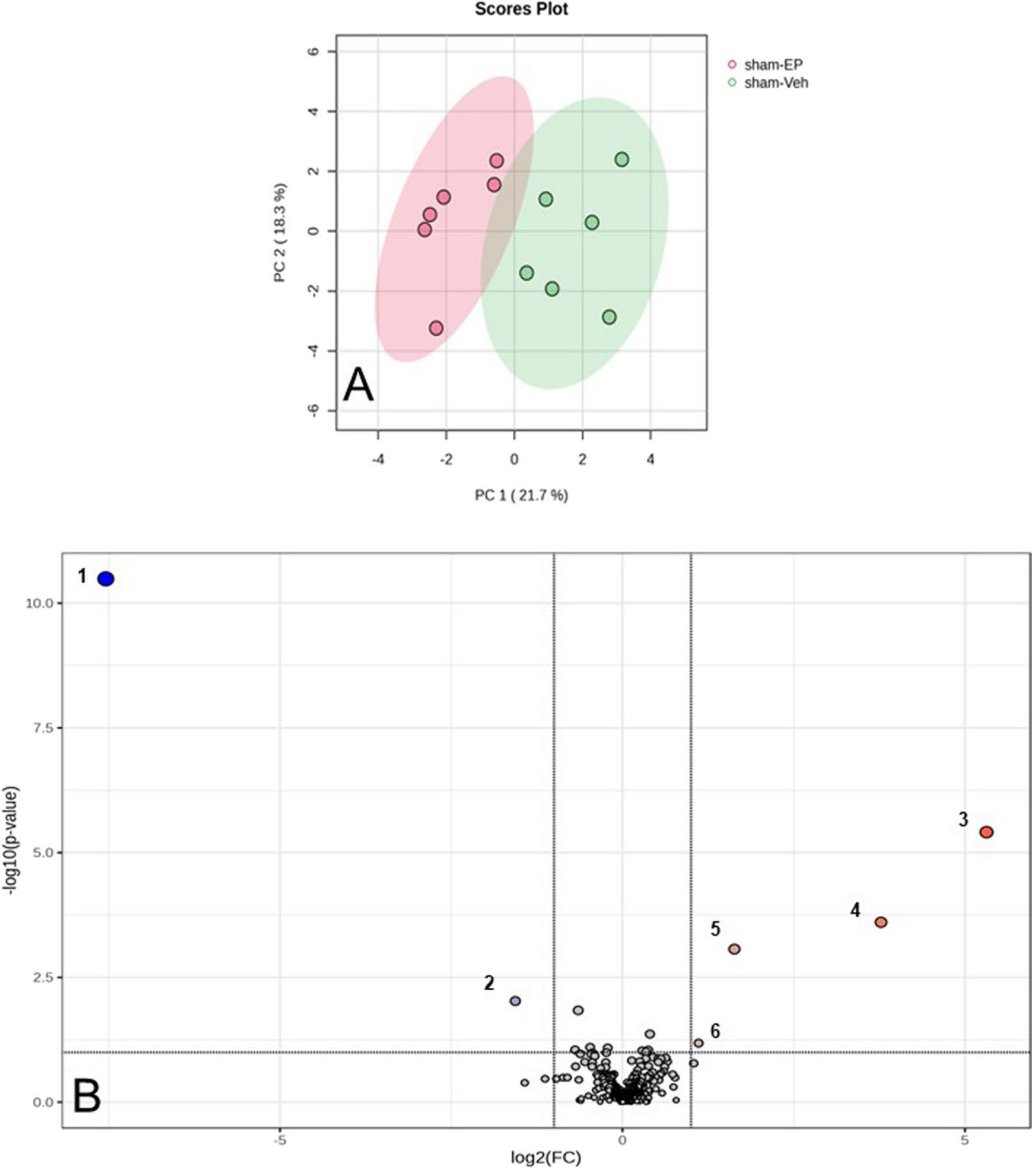
Effects of ethyl pyruvate treatment in the non-injured brain. **A** PCA score plot with 95% confidence ellipses showing a slight overlap in the metabolic profile of sham-EP and sham-Veh groups in PC1. **B** Volcano plot identified 6 metabolites that were significantly altered with EP treatment (fold change 2.0, *p* < 0.05). 1. glycerophosphocholine; 2. homeserine; 3. glycine; 4. glycylvaline; 5. glycylleucine; 6. 2-deoxyadenosine

PCA analysis shows that the metabolic profile of sham-EP and sham-Veh groups overlapped slightly in PC1, with sham-EP clustered towards the negative side of PC1 and sham-Veh clustering towards the positive side of PC1. PC1 and PC2 account for 21.7% and 18.3% of the total variance, respectively (Fig. 4A). EP treatment in the non-injured brain (sham-EP) resulted in a twofold concentration increase in 4 metabolites and a twofold decrease in concentration of 2 additional metabolites compared to the sham-Veh group (*p* < 0.05; Fig. 4B). Metabolites showing an increase belonged to the amino acid (glycine), peptide (glycylvaline, glycylleucine), and nucleotide (2ʹ deoxyadenosine) families. With metabolites decreasing in concentration belonging to the amino acid (homoserine) and lipid (glycerophosphorylcholine) families.

### Pathway analysis

#### Metabolic pathways affected by CCI injury

**Fig. 5:**
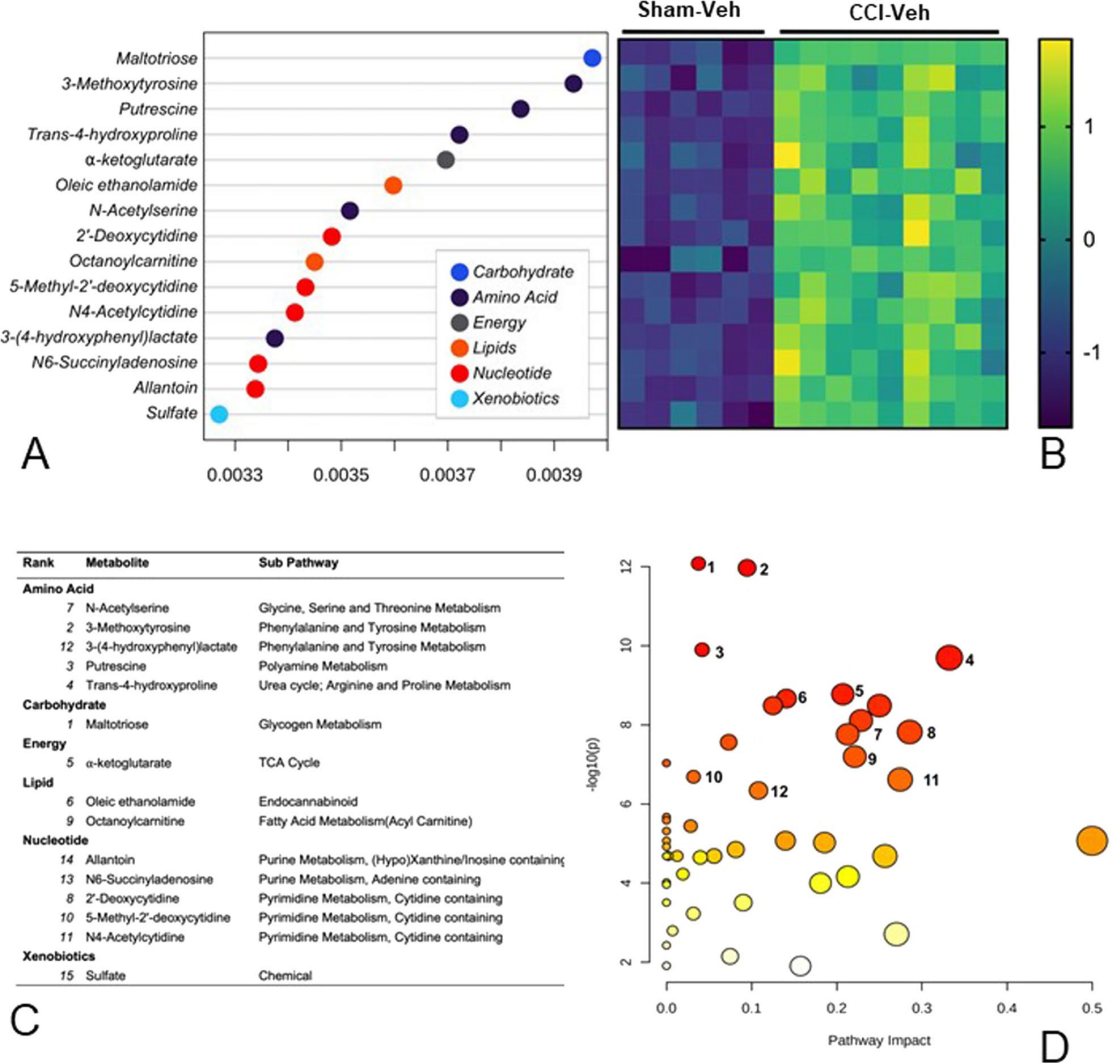
Random Forest and pathway analysis of the metabolite changes following CCI injury. **A** RFA variable importance plot identifying the 15 most impactful metabolites differentiating the CCI-Veh and sham-Veh groups. **B** The heatmap shows that these metabolites were increased in the CCI-Veh group compared to the sham-Veh group. **C** Super pathway and sub pathway families of the metabolites ranked in the variable importance plot. **D** Scatter plot of pathway impact versus pathway significance, where each node represents a significant metabolic pathway contributing to the difference between CCI-Veh and CCI-EP groups. The white to red node color scale indicates the p-value and node size reflects pathway impact score. Numbered pathways were selected based on having > 1 metabolite hit from the pathway analysis and > 0 pathway impact. 1. glutathione metabolism; 2. glycine, serine and threonine metabolism; 3. glycolysis/gluconeogenesis; 4. arginine and proline metabolism; 5. pyrimidine metabolism; 6. lysine degradation; 7. arginine biosynthesis; 8. taurine and hypotaurine metabolism; 9. alanine, aspartate, and glutamate metabolism; 10. butanoate metabolism; 11. cysteine and methionine metabolism; 12. tryptophan metabolism; 13. valine, leucine, and isoleucine degradation; 14. citrate (TCA) cycle; 15. amino sugar and nucleotide sugar metabolism

Random Forest analysis (RFA) was used to identify the metabolites that were most important in distinguishing the metabolic characteristics associated with CCI injury and EP effects. The variable importance plot identified 15 metabolites that significantly contributed to the model’s predictive accuracy in differentiating between the CCI-Veh and sham-Veh groups (Fig. 5A). The metabolites belong to several super metabolic families including carbohydrate, amino acid, energy, lipid, nucleotide, and xenobiotic metabolism (Fig. 5A, C). The heatmap shows that all fifteen metabolites were increased in the CCI-Veh group, as indicated by the transition from blue shades green (Fig. 5B). To evaluate which pathways were significantly altered by CCI injury, we combined the metabolites identified in the RFA (Fig. 5A, C) with the significant metabolites identified in the volcano plot (Fig. 4). Pathway analysis identified significant changes in 39 metabolic pathways (Fig. 5D, Holm *p* < 0.05).

**Fig. 6:**
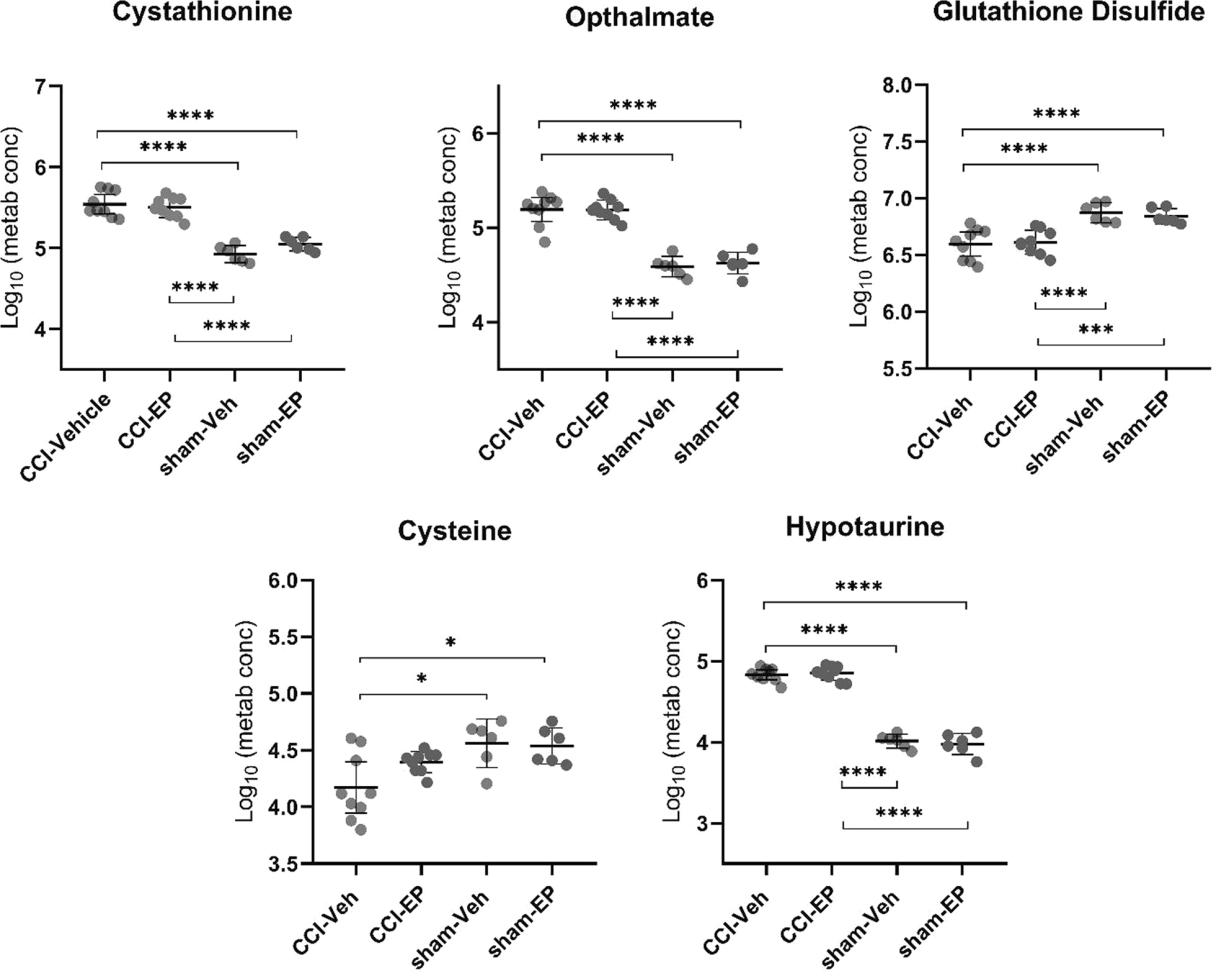
CCI-induced alterations in metabolites associated with thiol metabolism. Pathways significantly altered included glycine, serine and threonine metabolism, glutathione metabolism, taurine and hypotaurine metabolism, and cysteine and methionine metabolism. All four pathways have cysteine as one of their metabolic hits in the pathway analysis. Between group comparisons of the mean (± standard deviation) metabolite concentrations (Log_10_), using one way analysis of variance where *p < 0.05, ****p < 0.0001.

**Fig. 7:**
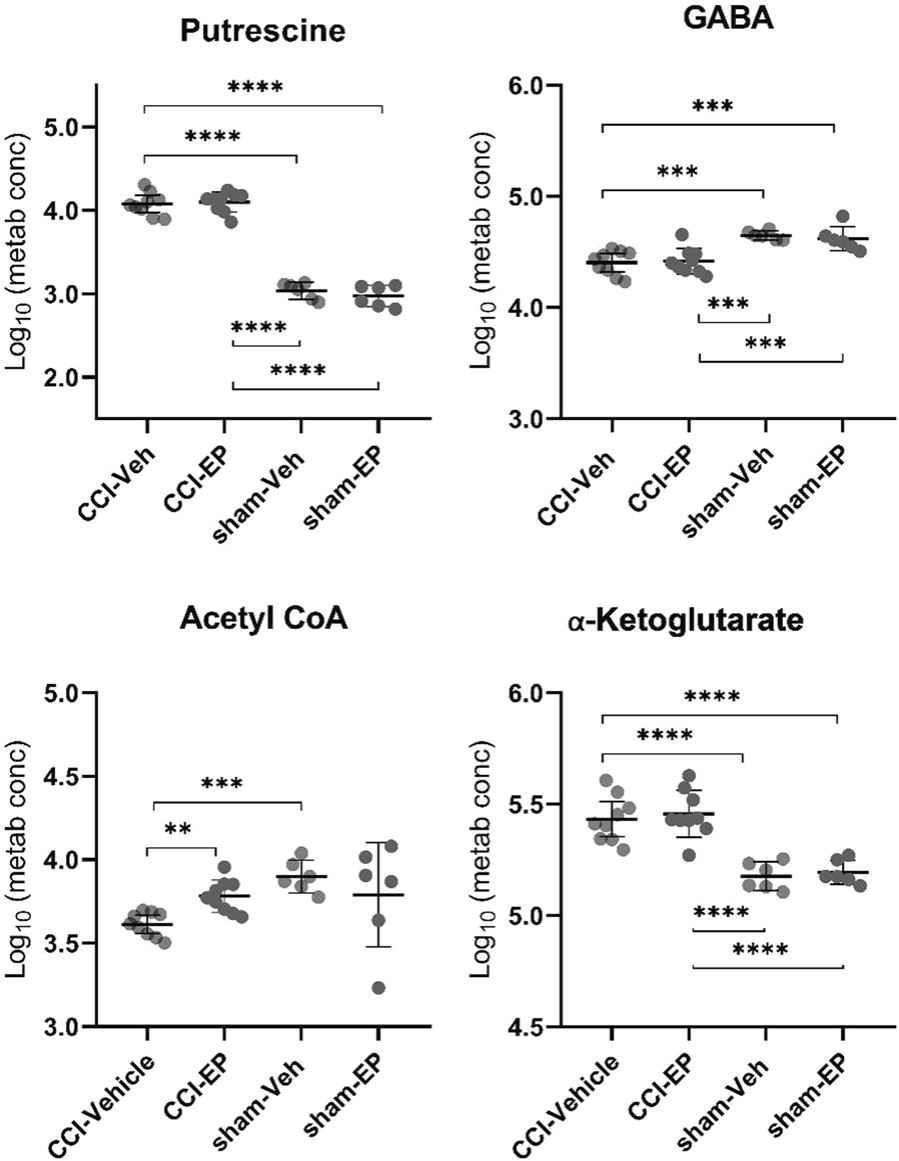
CCI-induced alterations in metabolites associated with GABA and TCA cycle intermediates. Significantly altered pathways included arginine and proline metabolism, alanine, aspartate and glutamate metabolism, and the TCA cycle. Between group comparisons of the mean (± standard deviation) metabolite concentrations (Log_10_), using one way analysis of variance where *p < 0.05, **p < 0.01, ***p < 0.001, ****p < 0.0001

**Fig. 8:**
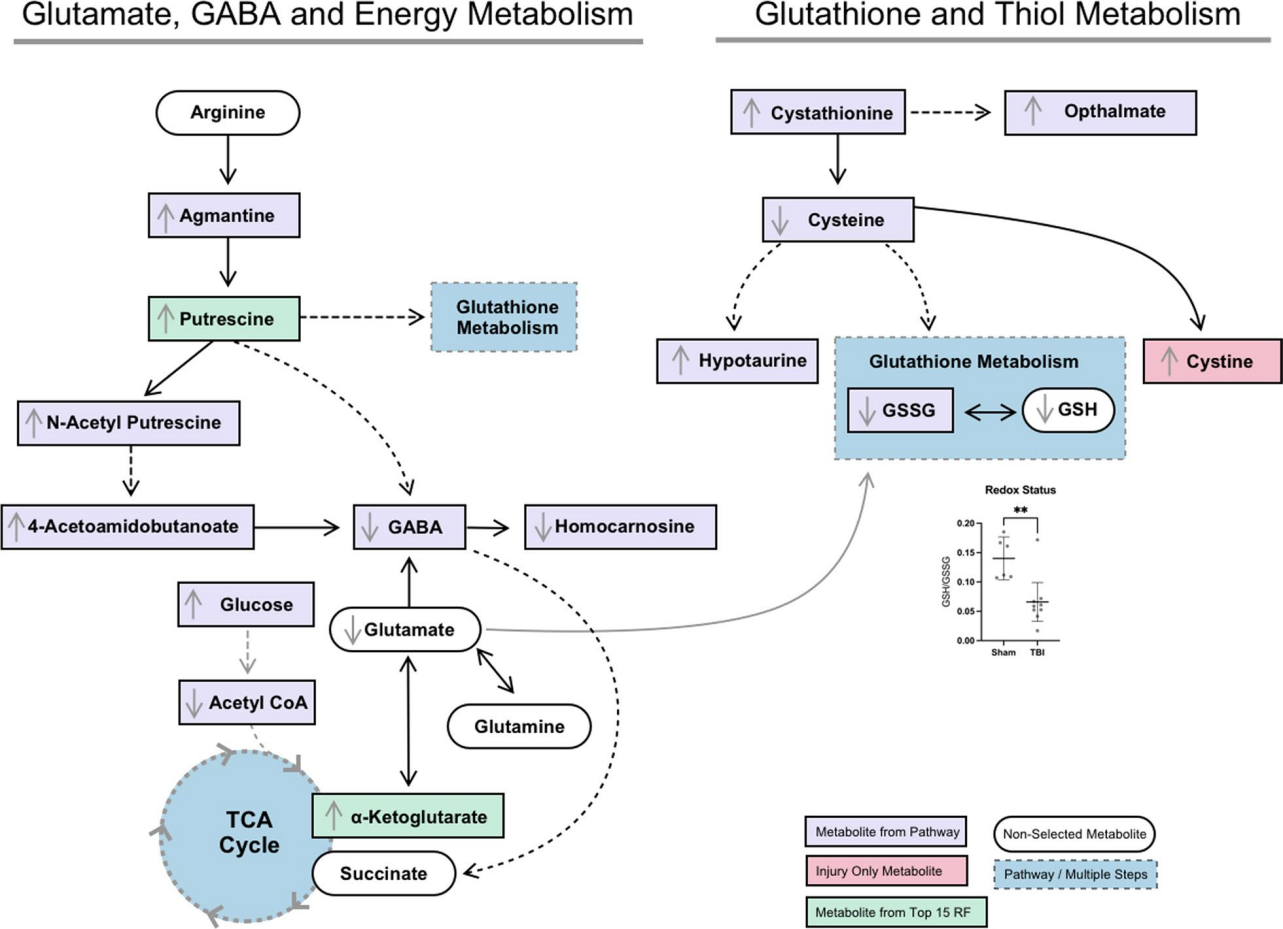
Summary of metabolic changes following acute experimental TBI. Redox status is significantly different between sham-Veh and CCI-Veh groups (t = 3.53), Student’s t-test, ** *p* < 0.005)

Nineteen of the most significantly impacted pathways were associated with a significant change in only one metabolite, as seen for ascorbate and aldarate metabolism, as well as pentose and glucuronate interconversions that were both driven by increases in the end-product glucuronate. An increase in the metabolite ⍺-ketoglutarate was associated with a change in both butanoate and arginine metabolism, while significantly altered sulfur metabolism was a result of increased sulfate. Pathways with significant alterations in multiple metabolites are numbered in Fig. 5D and presented in Table 1. These included pathways associated with glutathione, glycine/serine/threonine, glycolysis/gluconeogenesis, arginine/proline, lysine, pyrimidine, taurine/hypotaurine, alanine/aspartate/glutamate, butanoate, cysteine/methionine, and tryptophan metabolism. Of particular interest were the pathways associated with thiol (Fig. 6), GABA, and TCA cycle intermediate metabolism (Fig. 7). Glutathione, glycine/serine/threonine, taurine/hypotaurine and cysteine/methionine pathways all had cysteine as a metabolic hit (Fig. 6). Cysteine was significantly reduced in the CCI-Veh group compared to both the sham-Veh and sham –EP groups (Fig. 6, p < 0.05) and represents a decision point in metabolism as it can be converted into several products (Fig. 8). Along with cysteine, several other metabolites associated with thiol metabolism were altered (Fig. 6). Glutathione disulfide was significantly decreased compared to both the sham-Veh and sham-EP groups (*p* < 0.001) while cystathionine, opthalmate and hypotaurine were all increased in the CCI-Veh group 24 h following injury (*p* < 0.0001). Several pathways related to GABA and the TCA cycle were also altered, including arginine/proline, alanine/aspartate/glutamate, and TCA cycle intermediates (Fig. 7). Like cysteine, putrescine can be diverted to several different metabolic pathways (Fig. 8) and was found to be increased in the CCI-Veh group compared to sham-Veh and sham-EP groups (Fig. 7, *p* < 0.0001). While putrescine was increased, GABA was decreased in the CCI-Veh group compared to sham-Veh and sham-EP groups (Fig. 7, *p* < 0.0001). Related to the TCA cycle, acetyl CoA was significantly decreased compared to the sham-Veh group, while ⍺-ketoglutarate was increased following injury (Fig. 7, *p* < 0.001).

**Table 1:** Significantly enriched metabolic pathways following CCI injury

Enriched pathways	Holm	FDR	Impact	Metabolite hits
Glutathione metabolism	4.0E−11	2.6E−11	0.038	Glutathione disulfide; l-Cysteine; Acetyl-CoA; Putrescine
Glycine, serine and threonine metabolism	5.0E−11	2.6E−11	0.095	Betaine; Guanidinoacetate; l-Cystathionine; l-Threonine; l-Cysteine
Glycolysis/gluconeogenesis	5.8E−09	2.0E−09	0.042	Acetyl-CoA; beta-d-Glucose
Arginine and proline metabolism	9.0E−09	2.4E−09	0.332	Guanidinoacetate; GABA; Agmatine; Putrescine; *N*-Acetylputrescine; Hydroxyproline; l-Proline; 4-Acetamidobutanoate; Homocarnosine;
Pyrimidine metabolism	7.4E−08	1.6E−08	0.207	dCMP; Deoxycytidine; Thymidine; Thymine; *N*-Carbamoyl-l-aspartate; Orotate
Lysine degradation	9.3E−08	1.7E−08	0.141	L-2-Aminoadipate; l-Pipecolate; Acetyl-CoA
Arginine biosynthesis	3.1E−07	4.1E−08	0.228	l-Citrulline; ⍺-Ketoglutarate
Taurine and hypotaurine metabolism	5.9E−07	7.3E−08	0.286	l-Cysteine; Hypotaurine
Alanine, aspartate and glutamate metabolism	2.3E−06	2.3E−07	0.221	*N*-Acetyl-l-aspartate; l-Alanine; GABA; Citrate; *N*-Carbamoyl-l-aspartate; ⍺-Ketoglutarate
Butanoate metabolism	7.0E−06	6.6E−07	0.032	Acetyl-CoA; GABA; ⍺-Ketoglutarate
Cysteine and methionine metabolism	7.9E−06	7.2E−07	0.274	l-Cystathionine; l-Cysteine; Ophthalmate
Tryptophan metabolism	1.5E−05	1.3E−06	0.108	5-Hydroxyindoleacetate; l-Kynurenine; Acetyl-CoA
Valine, leucine and isoleucine degradation	1.0E−04	8.7E−06	0.028	Acetyl-CoA; l-Valine; l-Isoleucine
Citrate cycle (TCA cycle)	2.3E−04	1.8E−05	0.186	⍺-Ketoglutarate; Acetyl-CoA; citrate
Amino sugar and nucleotide sugar metabolism	3.1E-−04	2.5E−05	0.081	d-Mannose 6-phosphate; beta-d-Fructose

The top fifteen significantly enriched metabolic pathways associated with the metabolic differences between CCI-Veh and CCI-sham groups. Pathways that were selected include those with > 1 metabolic hit and > 0 pathway impact. Both acetyl-CoA and l-cysteine were hits in multiple enriched pathways, both being reduced following injury

#### Metabolic pathways affected by EP treatment in the injured brain

**Fig. 9:**
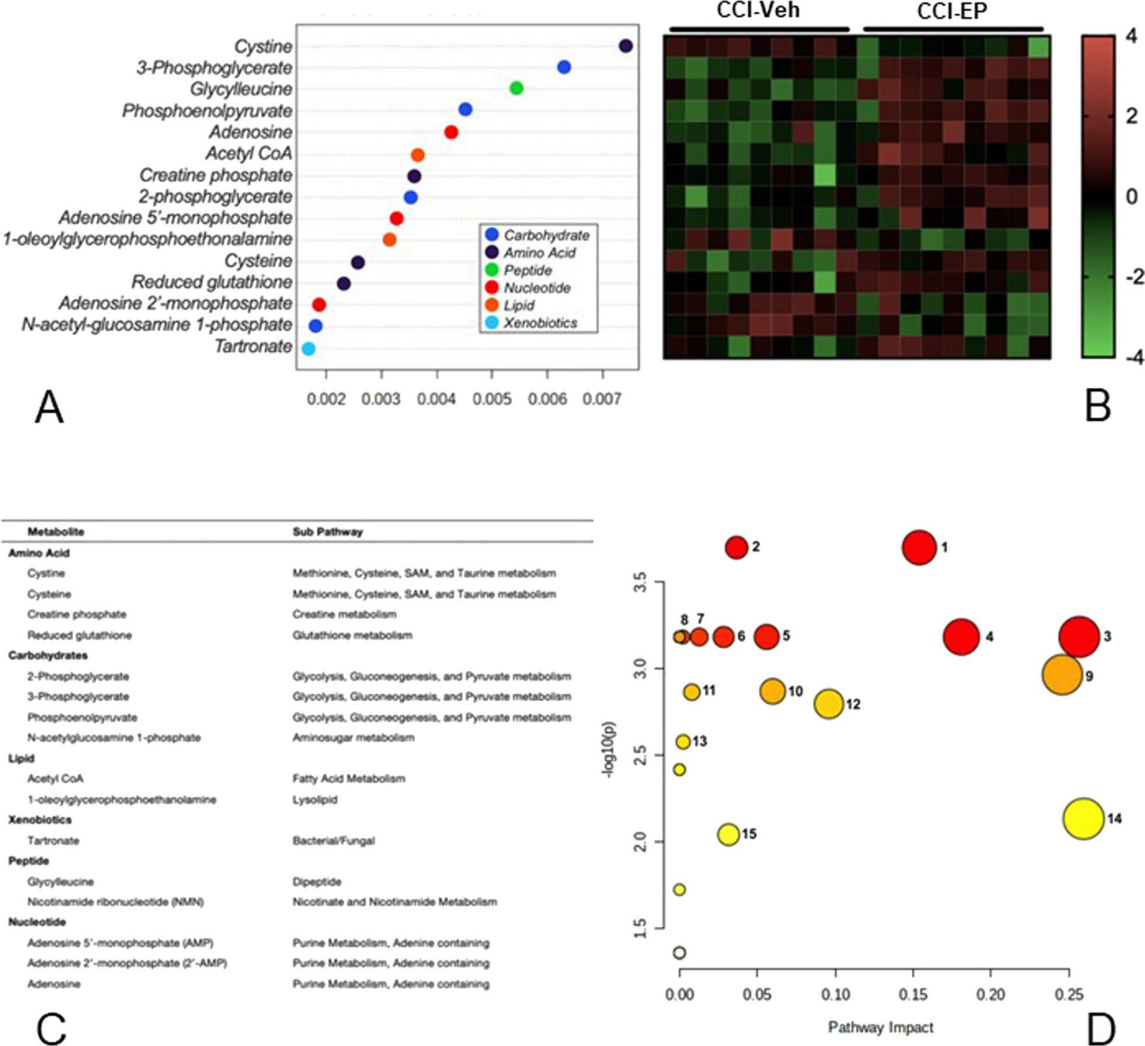
Random Forest and pathway analysis of metabolic changes in the injured brain following treatment with EP. **A** Random Forest analysis variable importance plot and (**B**) heatmap of the 15 most impactful metabolites separating the CCI-EP and CCI-Veh groups. **C** Super pathway and sub pathway designation of metabolites identified in the variable importance plot. **D** Scatter plot of pathway significance versus pathway impact identifying the metabolic pathways contributing to the difference between CCI-EP and CCI-Veh groups, with white to red node color indicating largest (red) to smallest (white) p-value. Node size indicates pathway impact score. Criteria for selecting pathways was based on > 1 metabolite hit in the pathway analysis and a > 0 impact score. 1. pyruvate metabolism; 2. citrate (TCA) cycle; 3. fatty acid elongation; 4. fatty acid degradation; 5. beta-alanine metabolism; 6. valine, leucine, and isoleucine degradation; 7. propanoate metabolism; 8. fatty acid biosynthesis; 9. glycolysis/gluconeogenesis; 10. purine metabolism; 11. glyoxylate and dicarboxylate metabolism; 12. cysteine and methionine metabolism; 13. glycine, serine, and threonine metabolism; 14. glutathione metabolism; 15. nicotinate and nicotinamide metabolism

RFA identified the top 15 metabolites that significantly contributed to the separation between the CCI-Veh and CCI-EP groups, which belong to the amino acid, carbohydrate, lipid, nucleotide, peptide, and xenobiotic super families (Fig. 9A, C). The heatmap shows that 66% of the identified metabolites were increased in the CCI-EP group compared to the CCI-Veh group (Fig. 9B). Pathway analysis of the combined metabolites identified in the variable importance plot and the significant metabolites identified in the volcano plot showed significant changes in 18 metabolic pathways (Holm *p* < 0.05, Table 2, Fig. 9D).

**Table 2:** Significantly enriched metabolic pathways following EP treatment in the CCI injured brain

Enriched Pathways	Holm	FDR	Impact	Metabolite hits
Pyruvate metabolism	5.6E−03	1.3E−03	0.154	Acetyl-CoA; phosphoenolpyruvate
Citrate cycle (TCA cycle)	5.6E−03	1.3E−03	0.037	Acetyl-CoA; phosphoenolpyruvate
Fatty acid elongation	1.7E−02	1.3E−03	0.037	Acetyl-CoA
Fatty acid degradation	1.7E−02	1.3E−03	0.181	Acetyl-CoA
Beta-alanine metabolism	1.7E−02	1.3E−03	0.056	Acetyl-CoA
Valine, leucine and isoleucine degradation	1.7E−02	1.3E−03	0.028	Acetyl-CoA
Propanoate metabolism	1.7E−02	1.3E−03	0.013	Acetyl-CoA
Fatty acid biosynthesis	1.7E−02	1.3E−03	0.002	Acetyl-CoA
Glycolysis/gluconeogenesis	1.7E−02	2.0E−03	0.246	Acetyl-CoA; 2-Phospho-d-glycerate; Phosphoenolpyruvate
Purine metabolism	1.8E−02	2.3E−03	0.060	Adenosine 5ʹ-monophosphate; adenosine
Glyoxylate and dicarboxylate metabolism	1.8E−02	2.3E−03	0.008	Acetyl-CoA; 2-Phospho-d-glycerate
Cysteine and methionine metabolism	1.8E−02	2.5E−03	0.096	l-Cystine; l-Cysteine
Glycine, serine and threonine metabolism	2.6E−02	3.9E−03	0.002	2-Phospho-d-glycerate; l-Cysteine
Glutathione metabolism	5.1E−02	9.4E−03	0.260	2-Phospho-d-glycerate; l-Cysteine
Nicotinate and nicotinamide metabolism	5.4E−02	1.1E−02	0.032	Nicotinamide d-ribonucleotide

The top fifteen significantly enriched metabolic pathways associated with the metabolic differences between CCI-Veh and CCI-EP groups. Pathways that were selected include those with > 1 metabolic hit and > 0 pathway impact score. Table sorted by FDR value. Acetyl-CoA is a key metabolite impacted in multiple pathways

Pyruvate metabolism and the TCA cycle pathways were the most significantly altered pathways following EP treatment in the injured brain (Fig. 9D; Table 2). A large portion of the significant metabolic pathways had only one metabolic hit, defined as a significantly altered metabolite in the pathway of interest, as seen for fatty acid elongation and degradation, beta-alanine metabolism, valine, leucine and isoleucine degradation, propanoate metabolism, and fatty acid biosynthesis. These enriched pathways were driven by changes in acetyl-CoA, a product of pyruvate metabolism. Interestingly, glutathione metabolism had the greatest pathway impact score and consisted of metabolite hits in glutathione, cysteine, and acetyl-CoA.

**Fig. 10:**
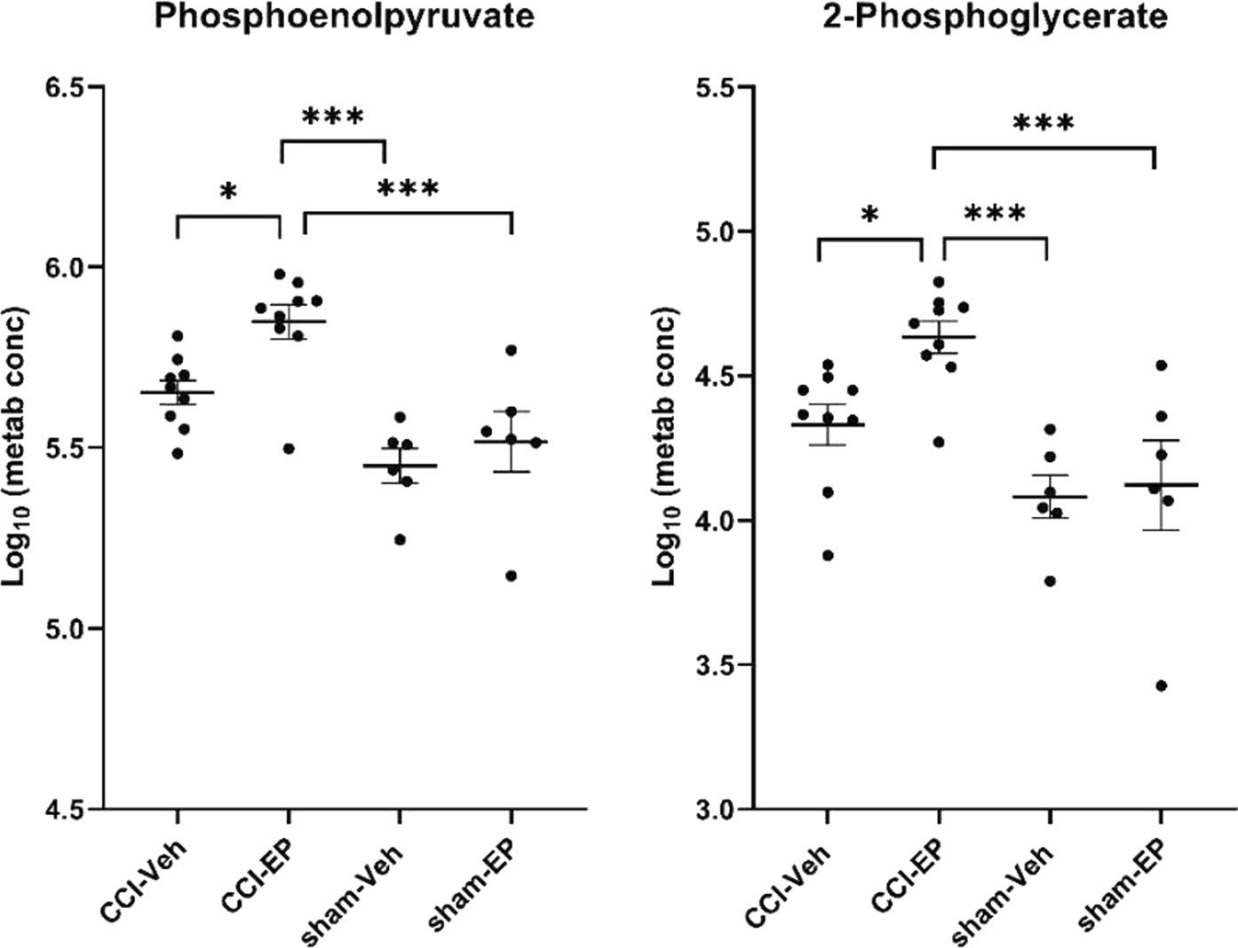
Significantly altered metabolites associated with energy metabolism following EP treatment in the injured brain. Significantly altered pathways following EP treatment included pyruvate metabolism, the TCA cycle, and glycolysis/gluconeogenesis. Phosphoenolpyruvate was a metabolic hit in all three pathways, and 2-phosphoglycerate was a metabolic hit in glycolysis/gluconeogenesis. Between group comparisons of the mean (± standard deviation) metabolite concentrations (Log_10_), using one way analysis of variance where *p < 0.05, ***p < 0.001

**Fig. 11:**
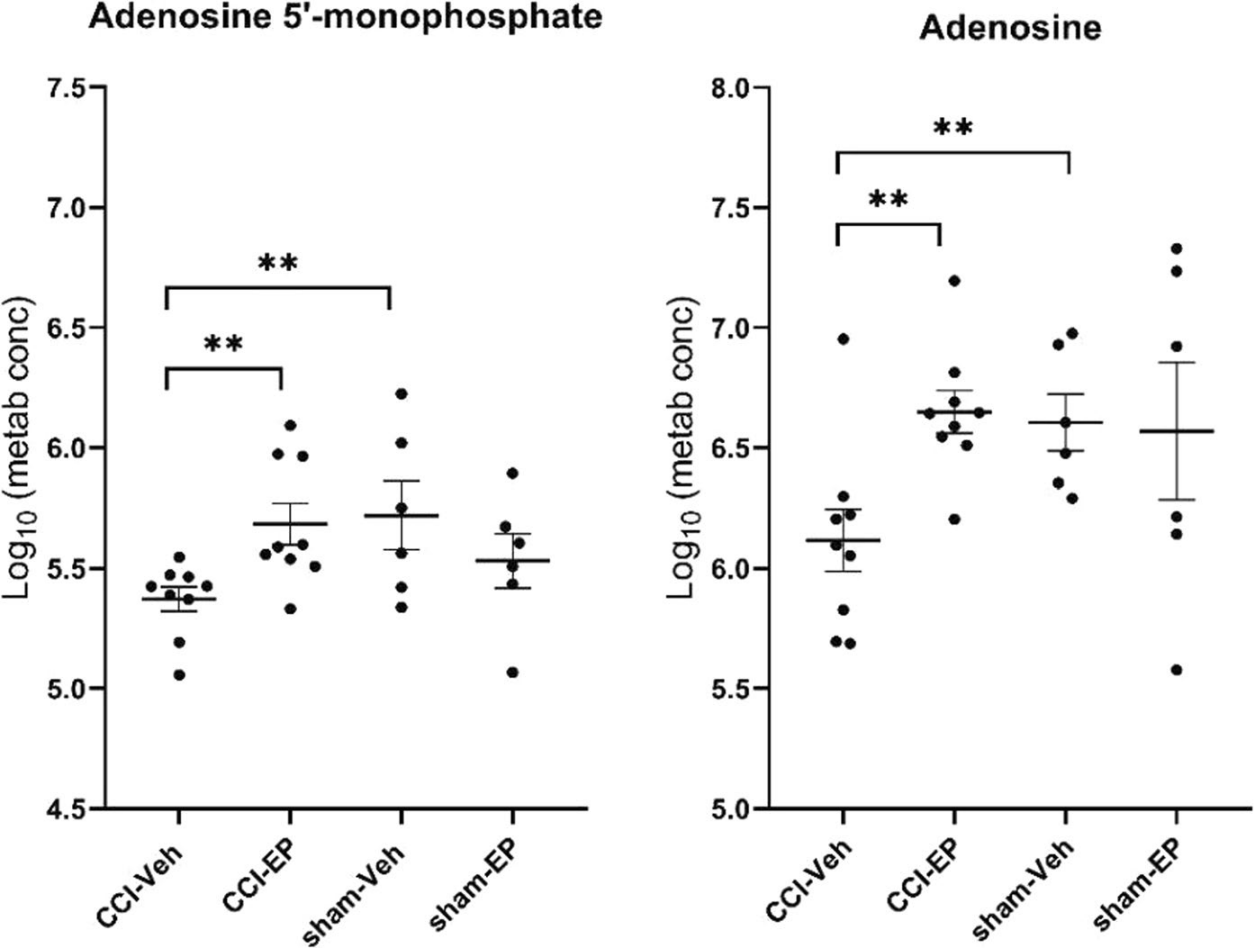
Significantly altered metabolites associated with purine metabolism following EP treatment in the injured brain. Adenosine 5ʹ-monophosphate and adenosine were significant metabolic hits in the pathway of purine metabolism. Between group comparisons of the mean (± standard deviation) metabolite concentrations (Log_10_) using one way analysis of variance where **p < 0.01

**Fig. 12:**
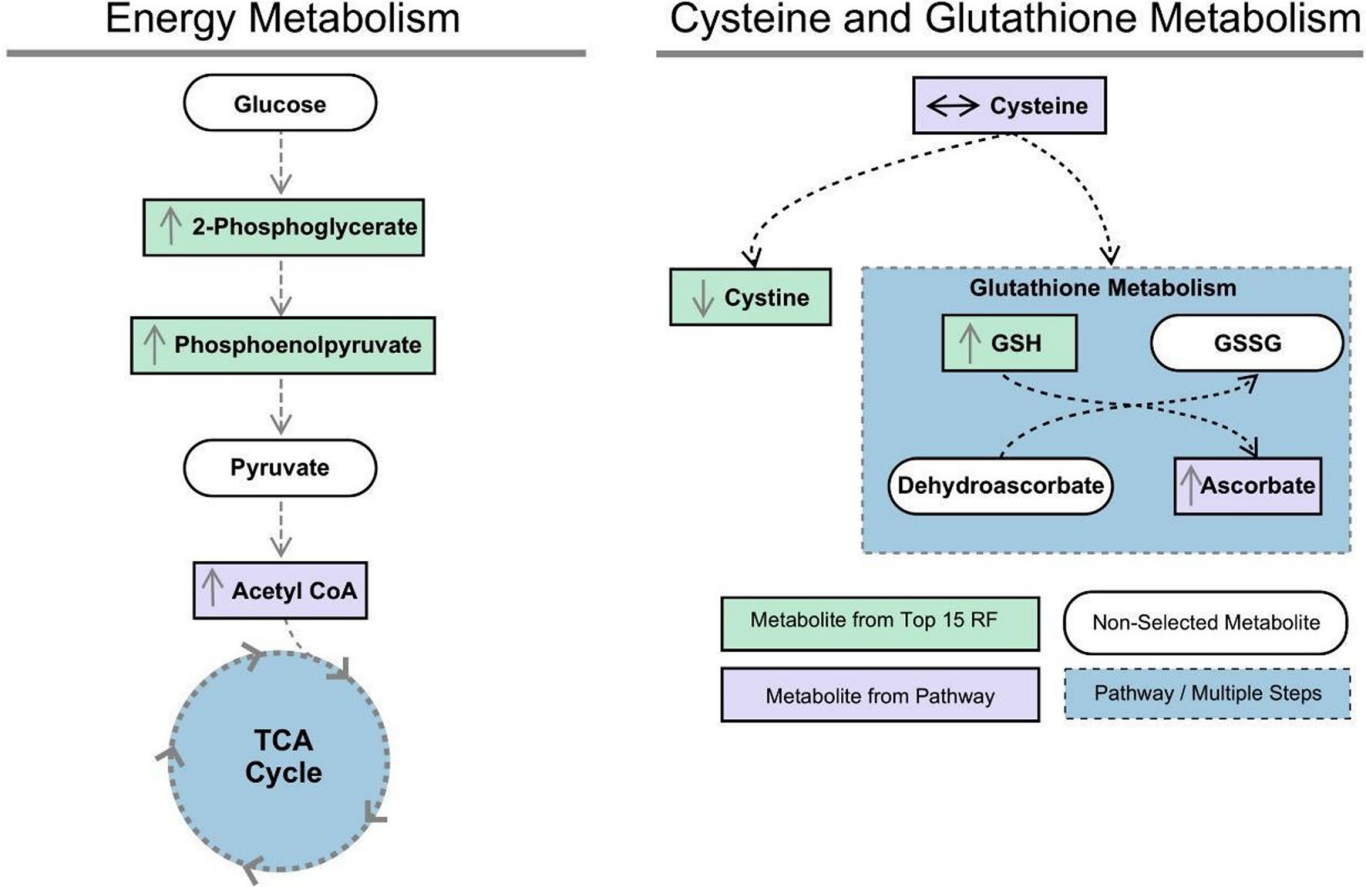
Summary of metabolic changes following EP treatment in the injured brain

In the CCI-EP group, there was a significant increase in acetyl-CoA and phosphoenolpyruvate levels compared to the CCI-Veh group within the pyruvate metabolism, TCA cycle, and glycolysis/gluconeogenesis pathways (*p* < 0.05, Figs. 7, 10). Specifically, for the glycolysis/gluconeogenesis pathway, 2-phosphoglycerate levels were significantly higher in the CCI-EP group compared to the CCI-Veh, sham-Veh, and sham-EP groups (*p* < 0.01, Fig. 10). Acetyl-CoA was also significantly elevated when compared to the CCI-Veh (*p* < 0.01) group but did not reach significance compared to the sham-EP group (Fig. 7). In the purine metabolism pathway, both adenosine 5ʹ-monophosphate and adenosine showed significant increases in the CCI-EP group relative to CCI-Veh (*p* < 0.01, Fig. 11). In the glutathione pathway, glutathione exhibited lower levels when compared to the sham-Veh and sham-EP groups (*p* < 0.001, Fig. 6). A summary of the metabolic pathways altered by EP treatment in the injured brain is depicted (Fig. 12).

#### Metabolic pathways affected by EP treatment in the non-injured brain.

**Fig. 13:**
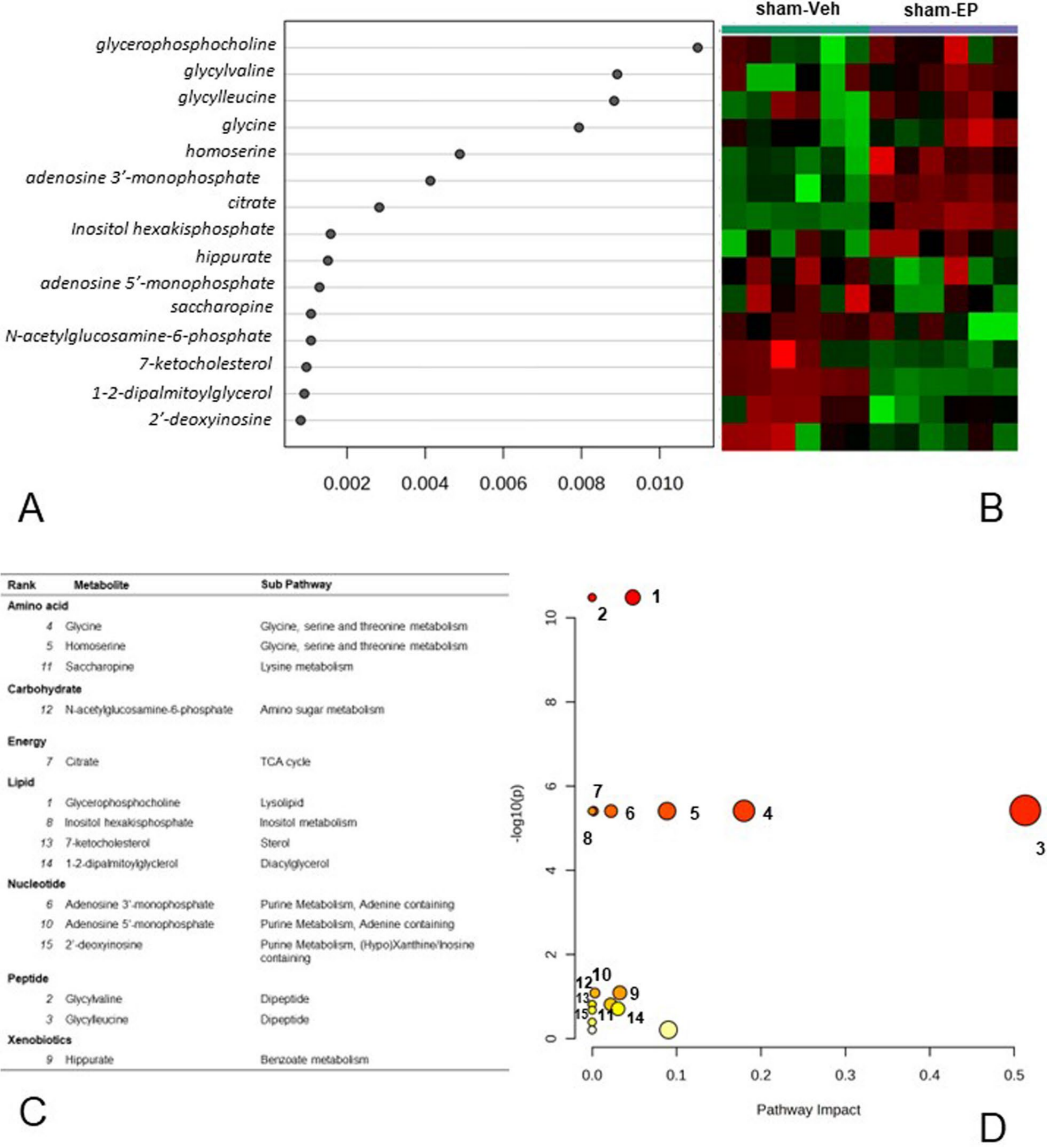
Random Forest and pathway analysis of metabolic changes in the non-injured brain following treatment with EP. **A** Random Forest analysis variable importance plot and (**B**) heatmap of top 15 metabolites separating the sham-Veh and sham-EP groups. **C** Super pathway and sub pathway of metabolites identified in the RFA. **D** Scatter plot of pathway significance versus pathway impact identifying the significantly impacted metabolic pathways contributing to the difference between sham-Veh and sham-EP groups. Pathways are sorted based on p-value, with white to red node color indicating largest (red) to smallest (white) p-value. Node size indicates pathway impact score. Criteria for selecting pathways was based on > 1 metabolite hit and > 0 impact score. 1. glycerophospholipid metabolism; 2. glycine, serine, threonine metabolism; 3. glyoxylate and dicarboxylate metabolism; 4. glutathione metabolism; 5. primary bile acid biosynthesis; 6. lipoic acid metabolism; 7. purine metabolism; 8. pyrimidine metabolism; 9. cysteine and methionine metabolism; 10. sphingolipid metabolism; 11. lysine degradation; 12. Inositol phosphate metabolism; 13. valine, leucine and isoleucine biosynthesis; 14. citrate cycle (TCA cycle); 15. alanine, aspartate and glutamate metabolism

RFA of the sham-Veh and sham-EP groups identified the top 15 metabolites separating the two sham groups which belonged to the amino acid, carbohydrate, energy, lipid, nucleotide, and peptide super metabolic families (Fig. 13A, C). The heatmap shows that many of the top 8 metabolites were increased in the sham-EP group compared to the sham-Veh group (Fig. 13B).

**Fig. 14:**
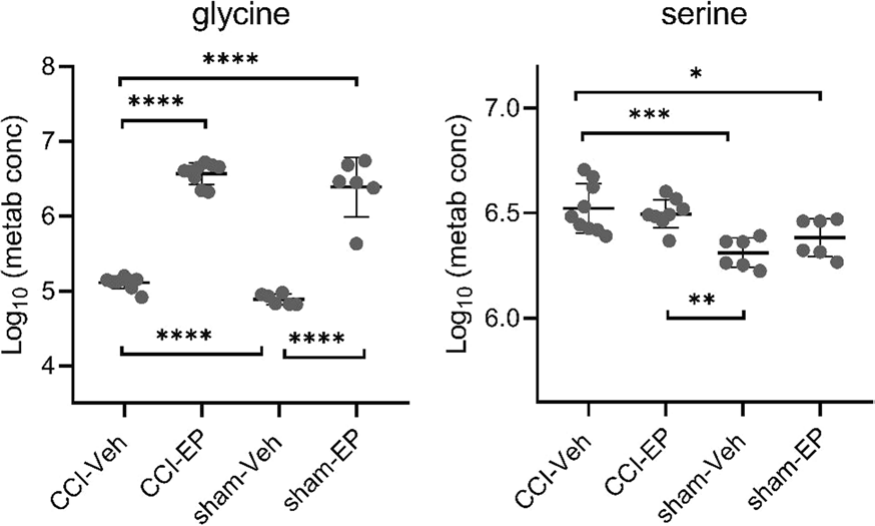
Significantly altered metabolites associated with glycine, serine, and threonine metabolism following EP treatment in the non-injured brain. Glycine and serine were significant metabolic hits in six of the most enriched metabolic pathways differentiating sham-Veh from sham-EP group. Between group comparisons of the mean (± standard deviation) metabolite concentrations (Log_10_) using one way analysis of variance where *p < 0.05, **p < 0.01, ****p < 0.0001

In the non-injured brain, glycerophospholipid and glycine, serine, and threonine metabolism pathways were the most significantly impacted pathways following EP treatment (Table 3). Six of the most enriched pathways were driven by changes in glycine and serine (Table 3). In the sham-EP group, there was a significant increase in glycine compared to the sham-Veh and CCI-Veh groups (*p* < 0.0001, Fig. 14). In contrast, there was no difference in glycine concentration between sham-EP and CCI-EP, strongly suggesting a purely EP effect (Fig. 14). Although highly impactful in differentiating between the sham-Veh and sham-EP groups, the serine concentration was not significantly elevated in the sham-EP group compared to the sham-Veh group (Fig. 14). However, serine was significantly elevated in the CCI-Veh and CCI-EP groups compared to either sham group suggesting an injury effect (Fig. 14).

**Table 3:** Significantly enriched metabolic pathways following EP treatment in the non-injured brain

Enriched pathways	Holm	FDR	Impact	Metabolite hits
Glycerophospholipid metabolism	5.88E−10	2.94E−10	0.04814	Glycerophosphocholine
Glycine, serine and threonine metabolism	5.98E−05	8.75E−06	0.51302	Glycine, serine, threonine
Glyoxylate and dicarboxylate metabolism	5.98E−05	8.75E−06	0.1799	Glycine, serine, citrate
Glutathione metabolism	5.98E−05	8.75E−06	0.08873	Glycine
Primary bile acid biosynthesis	5.98E−05	8.75E−06	0.02239	Glycine
Lipoic acid metabolism	5.98E−05	8.75E−06	0.0017	Glycine
Purine metabolism	0.80989	0.14747	0.03277	Adenosine-5ʹ-diphosphate, deoxyadenosine, deoxyinosine
Pyrimidine metabolism	0.80989	0.14747	0.0033	Deoxycytidine
Cysteine and methionine metabolism	1	0.21211	0.02184	Serine
Lysine degradation	1	0.24962	0.03067	Saccaropine
Citrate cycle (TCA cycle)	1	0.61551	0.09038	Citrate

The top 11 significantly enriched metabolic pathways associated with the metabolic differences between sham-Veh and sham-EP groups. Pathways that were selected include those with > 1 metabolic hit and > 0 pathway impact score. Glycine and serine are key metabolites impacted in multiple pathways

In the data filtering process, metabolites with > 50% missing values in any group (*n* = 49) were excluded from analysis (see “Methods”). However, some of these metabolites may be informative to TBI pathophysiology. In a separate analysis, we selected metabolites that exhibited less than 20% missing values in the CCI-Veh group and had greater than 80% missing values in the sham-Veh group for examination. Injury-induced metabolites were classified as amino acids, carbohydrates, cofactors and vitamins, lipids, nucleotides, and xenobiotics, with the greatest number of metabolites belonging to the amino acid and lipid superfamilies (Additional file 1).

## Discussion

The primary aims of this study were to examine the early metabolic alterations post-CCI and assess the effects of EP treatment in the injured and non-injured brain by analyzing specific metabolic pathways and related metabolites.

### Oxidative stress and antioxidant responses post-TBI

Due to its high oxygen consumption, the brain is particularly vulnerable to ROS generation, and this susceptibility is exacerbated by mitochondrial dysfunction, a common consequence of TBI, leading to rapid accumulation of excessive ROS [32]. To compensate for the accumulating ROS, antioxidants are needed. Our study demonstrated acute alterations in metabolites associated with sulfur-containing amino acid metabolism, including cysteine and hypotaurine, which are directly associated with the synthesis of antioxidants [33]. Of particular interest is the reduced level of cysteine following injury. Cysteine is synthesized from cystathionine and is tightly regulated due to its toxic effects [34]. Cysteine can be used in multiple downstream pathways, several of which were identified by pathway analysis including glycine, serine and threonine metabolism, taurine and hypotaurine metabolism, and glutathione metabolism. In our selected metabolites of interest, these downstream products included hypotaurine, opthalmate and glutathione disulfide. Interestingly, on our list of injury-induced only metabolites, there was also an increase in cystine which is formed by the autooxidation of cysteine. Consistent with our knowledge of the acute injury response, cystine, glutathione and opthalmate are all indicators of increased oxidative stress [35]. Interestingly, opthalmate has been proposed as a biomarker for hepatic glutathione depletion [36], which is consistent with our data, where opthalmate showed an inverse relationship with glutathione.

### Glutathione metabolism and redox regulation

Glutathione (GSH) is a tripeptide composed of glutamate, cysteine, and glycine. As a potent antioxidant, glutathione utilizes the sulfhydryl group of its cysteine to reduce ROS, itself dimerizing and becoming an oxidized glutathione disulfide (GSSG). Glutathione synthesis is regulated by the availability of cysteine, the rate-limiting precursor, as well as non-allosteric feedback competitive inhibition by glutamate [37–41]. The antioxidant and regenerative capacity of glutathione makes it an essential regulator of the redox status [42]. In our study, both GSH and GSSG were reduced 24 h after injury with a decrease in overall redox status, supporting a state of oxidative stress. The overall reduction in glutathione observed in our study might stem from a diminished availability of cysteine or changes in glutamate release, which affect glutathione synthesis and increase its degradation [42]. These results suggest that the injury-induced decrease in glutathione could exacerbate the oxidative stress environment, thereby amplifying neuronal damage and impairing recovery processes. Additionally, the diminished glutathione pool can result in reduced detoxification of ROS, potentially leading to higher levels of lipid peroxidation and protein oxidation, both of which have been observed in TBI patients [43]. This indicates that glutathione depletion plays a critical role in the progression of secondary injury post-TBI.

Additionally, excreted GSH from astrocytes being converted to cysteine-glutathione disulfide could further contribute to the observed lower levels of both cysteine and glutathione disulfide [44]. Astrocytes are known to release GSH as a protective measure, but this leads to the depletion of their intracellular GSH pools, which could also compromise their own antioxidant defenses, further weakening the neuroprotective response [45]. Moreover, studies suggest that the ratio of reduced glutathione (GSH) to oxidized glutathione (GSSG) is a key indicator of cellular redox state, and a significant drop in this ratio is linked with enhanced cellular apoptosis and inflammation [46]. Therefore, the reduction in the GSH ratio in our study further reinforces the hypothesis that TBI induces a severe oxidative environment, contributing to the observed neuronal cell death.

### Role of cystine in the TBI-induced metabolome

In our study, cystine was an injury-induced metabolite that was detected in only one sham sample. Similar to GSH/GSSG, the ratio between cysteine and cystine is decreased in states of oxidative stress as GSH and cysteine become oxidized [35]. Interestingly, while GSSG and cystine do share a common precursor, cysteine, there is independent regulation of oxidation of these two molecules [47]. Consistent with our metabolic data, in human plasma, the cysteine/cystine redox state was more oxidized than GSH/GSSG [47]. While no study has directly evaluated the role of cystine following TBI, it is uncertain if cystine is a marker of oxidative state and inflammation or an instigator of the inflammatory process.

### Taurine and hypotaurine metabolism post-injury

Along with the glutathione metabolism pathway, cysteine is also involved in the taurine and hypotaurine pathway as indicated by our pathway analysis. In the taurine and hypotaurine metabolism pathway, decreases in cysteine and concomitant increases in hypotaurine were the two hits in the pathway analysis. The metabolism of taurine and hypotaurine was also an enriched pathway in a previous study on hippocampal metabolic changes following injury [48]. The study found taurine and hypotaurine metabolism in the hippocampus to be a significantly altered pathway following CCI at day three but not at 24 h after injury. This difference could be a result of several factors including distance from injury site, brain region, and difference in feature selection methods. Biochemically, the increased levels of hypotaurine could explain the decreased levels of cysteine and GSH, indicating a shift from GSH synthesis to hypotaurine synthesis. It is unclear if this is deleterious or compensatory, as little research is available on the role of hypotaurine following TBI. Hypotaurine is a sulfur-containing metabolite with a diverse set of functions which may include acting as an antioxidant, anti-inflammatory agent, osmolyte and modulator of neurotransmission [49–51]. As an antioxidant, hypotaurine is a scavenger for both hypochlorous acid (HOCl) and highly reactive hydroxyl radicals [52]. Hypotaurine has also been shown to inhibit GABA uptake in the choroid plexus as well as inhibit both taurine uptake and synthesis [53–55]. Along with modulation of GABA signaling, hypotaurine may also modulate glycinergic neurotransmission [50]. While the effects of hypotaurine are diverse, to our knowledge no study has evaluated the specific role of hypotaurine following TBI. Our study and metabolic studies completed by Zheng et al. have demonstrated hypotaurine as a metabolite of interest in the acute phase of injury, but it is unclear what role it plays in injury and repair [48, 56]. Our data continues to support the acute modulation of antioxidants as a target for treatment and provides metabolites that highlight the integration of antioxidants with amino acid metabolism.

### The role of carnitines in TBI recovery

The significance of carnitines in the context of TBI is recognized, yet studies addressing their elevation post-injury remain sparse. This study contributes to the field by identifying carnitines as part of the fifteen metabolites whose presence significantly increases in TBI cases compared to non-injured controls. This finding aligns with existing literature that hints at the complex role carnitines play in TBI pathology and recovery [57, 58]. For instance, studies have shown that L-carnitine treatment can improve neurobehavioral function and reduce cerebral edema in TBI patients [57]. Carnitine's crucial role in mitochondrial metabolism and its potential protective effects against mitochondrial dysfunction and excitotoxicity, as evidenced in a hypoxia–ischemia study, underscores its therapeutic potential [58]. Moreover, the observed decrease in plasma free carnitine concentrations in severe trauma and TBI patients indicates a metabolic disruption that could be critical to understanding and treating TBI [59]. Although this study contrasts that finding, it emphasizes carnitines' significant elevation in TBI, suggesting their key role in TBI's metabolic response and potential in recovery mechanisms. This necessitates further investigation into carnitine level changes post-TBI, their clinical implications, and therapeutic potential.

### Ethyl pyruvate-induced metabolic changes and neuroprotective mechanisms post-TBI

In the injured brain EP treatment induced only a small number of metabolic changes. Of note, we observed a decrease in cystine but only in the presence of injury (CCI-EP) and not in the non-injured brain (sham-EP). Cystine is also integral to the production of cysteine, which, in turn, supports glutathione synthesis [60]. EP's ability to reduce cystine post-TBI may suggest that it enhances the metabolic flux towards increased GSH production, thereby fortifying the brain's antioxidant defenses. Since cystine serves as a precursor to the antioxidant GSH, this finding would support an anti-inflammatory role for EP, specifically a reduction in free radicals after TBI [42, 61]. Moreover, the suppression of cystine levels could mitigate the availability of substrates required for further ROS production, potentially explaining EP's protective effect on neurons. This aligns with previous reports that demonstrate EP's ability to suppress oxidative stress markers and neuroinflammation, particularly by inhibiting ROS production through its role in scavenging free radicals [18, 62].

Ascorbate is an abundant water-soluble antioxidant that has been found to be notably decreased after TBI, functioning as a cofactor for reduction reactions [63]. In our study, the CCI-EP group had a significant increase in this compound after injury which further supports the antioxidant potential of EP. The ability of EP to increase ascorbate levels may be crucial in limiting oxidative damage post-TBI, as ascorbate is essential for regenerating GSH from its oxidized form, GSSG [64]. This interplay between ascorbate and GSH reinforces the potential of EP to restore antioxidant balance and reduce secondary injury caused by oxidative stress.

NMN, another significantly increased compound in our study, is a direct precursor to the compound nicotinamide adenine dinucleotide (NAD+) which helps mediate DNA repair by serving as a substrate for polyADP-ribose polymerase (PARP) [5, 65, 66]. Together, these findings support prior reports of the neuroprotective effects of EP treatment following TBI [6, 21], which our study shows can be attributed to, in part, by their antioxidant effects. Furthermore, the NAD+ increase mediated by NMN could also enhance mitochondrial function, as NAD+ plays a critical role in maintaining mitochondrial oxidative metabolism, which is often impaired following TBI [67]. By restoring NAD+ levels, EP may help sustain energy production, reduce apoptosis, and facilitate neuronal survival in the acute phase post-injury.

### The role of ethyl pyruvate in enhancing glycolytic flux and mitigating inflammation

The increase in 2-phosphoglycerate and phosphoenolpyruvate levels in the CCI-EP group could be partly explained due to a direct metabolism of EP into acetyl-CoA in the mitochondrial matrix by the pyruvate dehydrogenase complex, subsequently entering the citric acid cycle [68]. This process may help alleviate the energy deficit observed post-TBI, as traumatic injury is known to disrupt ATP production and mitochondrial function, leading to a metabolic crisis [69]. By supplying acetyl-CoA, EP could directly contribute to ATP synthesis via the citric acid cycle and oxidative phosphorylation, providing the neurons with the necessary energy to survive during the critical post-injury period. The observed increase in 2-phosphoglycerate and phosphoenolpyruvate, intermediates of glycolysis, could indicate enhanced glycolytic flux, providing more pyruvate for mitochondrial energy metabolism, potentially alleviating the energy crisis commonly observed after TBI.

Furthermore, EP has anti-inflammatory properties, as evidenced by its ability to downregulate pro-inflammatory cytokines and inhibit high-mobility group box 1 (HMGB1), a key mediator of inflammation [62]. Beyond modulating cytokine production, EP’s effect on mitochondrial metabolism is linked to the reduction of mitochondrial dysfunction—a key driver of inflammation following TBI [19, 62]. Mitochondrial dysfunction often leads to the release of mitochondrial damage-associated molecular patterns (DAMPs), such as mitochondrial DNA, which can further amplify inflammatory signaling [70]. EP may help restore mitochondrial integrity, thereby mitigating DAMP release and the associated inflammatory cascades.

Additionally, the increase in AMP in the CCI-EP group may drive anti-inflammatory signaling via an activation of AMP-activated protein kinase A (AMPK), which activates proteins such as Nrf2 and HO-1 that are involved in neuroprotection [69, 71, 72]. Furthermore, the adenosine 2A receptor (A_2A_R) is known to play a significant role in neuroinflammation [73]. The interaction between the A_2A_R and the NLRP3 inflammasome, and the subsequent modulation of neuroinflammation, parallels the anti-inflammatory effects observed with EP treatment [74]. Specifically, the subsequent elevation of AMP and adenosine levels after EP treatment in the injured brain may similarly modulate A_2A_R activity or other adenosine receptors, thereby influencing the nucleotide-binding oligomerization domain, leucine-rich repeat, and pyrin domain-containing protein 3 (NLRP3) inflammasome's assembly and activation, which is crucial for the innate immune response in the central nervous system post-TBI. Thus, our finding that EP treatment elevates both AMP and adenosine following a CCI injury not only contribute to the understanding of adenosine's neuroprotective mechanisms but also position EP as a promising anti-inflammatory therapeutic agent for TBI.

By reducing neuroinflammation, EP may indirectly influence metabolic pathways, including glycolysis, by stabilizing the cellular environment and allowing for more efficient energy production and utilization. This could explain the observed increases in glycolytic intermediates, as a less inflamed environment may enhance glycolytic enzyme activities, promoting the conversion of glucose to pyruvate.

## Conclusions

This study elucidates the distinctive metabolic profiles of injury-induced and EP-induced metabolites after TBI, highlighting the metabolic responses to injury and EP treatment. Our findings reveal significant alterations in metabolites linked to inflammation, energy metabolism, and neuroprotection post-injury, and how EP modulates pathways related to reducing inflammation and oxidative stress in the injured brain. Findings which support future studies to explore the therapeutic potential of targeting these affected metabolic pathways in clinical settings.

## Supplementary Information

Additional file 1: Injury-induced metabolites. Fifteen metabolites were classified as injury-induced metabolites. These metabolites were present in > 80% of the CCI animals but were present in < 20% of sham animals

## Data Availability

The datasets used and/or analyzed during the current study are available from the corresponding author on reasonable request.

## Abbreviations

AMP: Adenosine 5ʹ-monophosphate
BBB: Blood–brain barrier
CCI: Controlled cortical impact
EP: Ethyl pyruvate
GC-MS: Gas chromatography–mass spectrometry
GSH: Glutathione
GSSG: Glutathione disulfide
HMDB: Human metabolome database
NMN: Nicotinamide ribonucleotide
NMR: Nuclear magnetic resonance
NLRP3: Nucleotide-binding oligomerization domain, leucine-rich repeat and pyrin domain-containing protein3
PCA: Principal component analysis
TBI: Traumatic brain injury
TCA: Tricarboxylic acid
UPLC-MS/MS: Ultra performance liquid chromatography–tandem mass spectrometry
