# Supplementary material for: Metabolic-driven analytics of traumatic brain injury and neuroprotection by ethyl pyruvate
Source: J Neuroinflammation. 2024 Nov 14;21:294. doi: 10.1186/s12974-024-03280-8 (PMC11562096; doi:10.1186/s12974-024-03280-8)
Supplement: Supplementary file 1 — Additional file 1: Injury-induced metabolites. Fifteen metabolites were classified as injury-induced metabolites. These metabolites were present in > 80% of the CCI animals but were present in < 20% of sham animals [file 12974_2024_3280_MOESM1_ESM.docx]

|  | **Metabolite** | **Sub Pathway** |
| --- | --- | --- |
| **Amino Acid** | | |
|  | Dimethylglycine | Glycine, Serine and Threonine Metabolism |
|  | 1-Methylhistidine | Histidine Metabolism |
|  | 3-Methylglutarylcarnitine | Lysine Metabolism |
|  | Cystine | Methionine, Cysteine, SAM and Taurine Metabolism |
|  | p-Cresol sulfate | Phenylalanine and Tyrosine Metabolism |
|  | Indolelactate | Tryptophan Metabolism |
|  | Kynurenate | Tryptophan Metabolism |
| **Carbohydrate** | | |
|  | 2-3-Diphosphoglycerate | Glycolysis, Gluconeogenesis, and Pyruvate Metabolism |
| **Cofactors and Vitamins** | | |
|  | N1-Methyl-2-pyridone-5-carboxamide | Nicotinate and Nicotinamide Metabolism |
| **Lipid** | | |
|  | Decanoylcarnitine | Fatty Acid Metabolism (Acyl Carnitine) |
|  | Cis-4-decenoyl carnitine | Fatty Acid Metabolism (Acyl Carnitine) |
|  | 1-Stearoylglycerophosphoglycerol | Lysolipid |
| **Nucleotide** | | |
|  | 3-Ureidopropionate | Pyrimidine Metabolism, Uracil containing |
| **Xenobiotics** | | |
|  | 4-Ethylphenylsulfate | Benzoate Metabolism |
|  | 2-Aminophenol sulfate | Chemical |
